# A Case for Studying Naturalistic Eye and Head Movements in Virtual Environments

**DOI:** 10.3389/fpsyg.2021.650693

**Published:** 2021-12-31

**Authors:** Chloe Callahan-Flintoft, Christian Barentine, Jonathan Touryan, Anthony J. Ries

**Affiliations:** ^1^Humans in Complex System Directorate, United States Army Research Laboratory, Adelphi, MD, United States; ^2^Warfighter Effectiveness Research Center, United States Air Force Academy, Colorado Springs, CO, United States

**Keywords:** head mounted display, eye tracking, eye movement analysis, virtual reality, smooth pursuit

## Abstract

Using head mounted displays (HMDs) in conjunction with virtual reality (VR), vision researchers are able to capture more naturalistic vision in an experimentally controlled setting. Namely, eye movements can be accurately tracked as they occur in concert with head movements as subjects navigate virtual environments. A benefit of this approach is that, unlike other mobile eye tracking (ET) set-ups in unconstrained settings, the experimenter has precise control over the location and timing of stimulus presentation, making it easier to compare findings between HMD studies and those that use monitor displays, which account for the bulk of previous work in eye movement research and vision sciences more generally. Here, a visual discrimination paradigm is presented as a proof of concept to demonstrate the applicability of collecting eye and head tracking data from an HMD in VR for vision research. The current work’s contribution is 3-fold: firstly, results demonstrating both the strengths and the weaknesses of recording and classifying eye and head tracking data in VR, secondly, a highly flexible graphical user interface (GUI) used to generate the current experiment, is offered to lower the software development start-up cost of future researchers transitioning to a VR space, and finally, the dataset analyzed here of behavioral, eye and head tracking data synchronized with environmental variables from a task specifically designed to elicit a variety of eye and head movements could be an asset in testing future eye movement classification algorithms.

## Introduction

Understanding how the visual system operates in the natural environment is a fundamental goal of cognitive psychology and has consequences for a variety of other research fields such as human factors and advertising. The natural environment offers a complex and uncontrolled input of visual information, making it is difficult to isolate variables of interest and determine their effect on behavior. Alternatively, constrained laboratory experimentation offers precise control, while potentially limiting the generalizability to less confined environments. To this end, vision researchers have begun to strike a balance between the lab and real world by running experiments in virtual reality (VR) using head mounted displays (HMDs). Experimentation in VR enables research paradigms that allow for more naturalistic behavior in subjects, while still providing experimental control over stimulus presentation (Clay et al., 2019). For purposes of clarity, we refer to the three-dimensional virtual environment, the digital X, Y, Z space in which one can present stimuli, *via* game engines such as Unity or Unreal, as VR (Watson et al., 2019). HMD refers specifically to the video display worn on the head, where subjects are immersed in a 360° virtual environment. Using VR in combination with HMDs allows experimenters precise control over stimulus timing and location, while offering subjects superior (when compared to traditional computer monitor setups) depth perception, a wider field of view, and the ability to move the eyes and head as they would in the real world (see Discussion for a more nuanced discussion of the limitations of VR and HMDs).

Integrating eye tracking (ET) with HMDs has further extended the research potential for this technology (Jangraw et al., 2014). Eye tracking has been an essential component in understanding how the visual system acquires information from a scene to build our internal perception. Measuring where the eyes foveate in a scene has been demonstrated in VR with HMD systems (Clay et al., 2019) and has given insight into how scene gist influences eye fixations during search (Boettcher et al., 2018) as well as how bottom-up and top-down influences guide the deployment of attention (Anderson et al., 2015; Harada and Ohyama, 2019). How the eyes move around a scene also provides important insight into cognitive processes (Williams and Castelhano, 2019) as well as clinical applications (Baloh et al., 1975; Terao et al., 2017; Ward and Kapoula, 2020). However, the vast majority of this research has been performed using a camera-based eye tracker and a two-dimensional monitor, which restricts the space stimuli are presented in and the subsequent behavior they induce. Newer technologies such eye tracking enabled HMDs, in addition to eye tracking glasses (ETGs), provide access to similar data but with the added benefit of tracking gaze in a 360° environment.

A growing effort has been made to study vision using more naturalistic scenes (Henderson et al., 2007; Dorr et al., 2010; Wolfe et al., 2011; O'Connell and Chun, 2018). However, equally important to exploring vision in the context of natural input (i.e., real-world scenes), is to explore vision in tandem with natural movement. Both HMD with VR and ETGs offer the freedom to move the head and torso when viewing the environment. ETGs have the added benefit of also allowing the subject to walk around the environment unrestricted, whereas subjects are typically more limited in HMDs, having to rely on unnatural modes of transport such as teleportation to avoid collisions with physical objects and to maintain a position within the headset-tracking volume. However, HMDs do allow more natural movement on smaller scales (e.g., room-size) and ETGs do not control for stimulus presentation that can be variable and unpredictable in real environments and may be less viable in situations such as training, where *in situ* exposure could be dangerous and/or costly (e.g., a simulated battlefield). In either circumstance, the ability to quantify more complex and dynamic eye movement patterns observed is limited as the majority of classification algorithms were developed with static 2D stimuli, and do not generalized to naturalistic contexts (Agtzidis et al., 2020).

The current work uses a visual discrimination task with unrestricted eye and head movement. Elicited patterns of activity are then classified based on the thresholds of I-S^5^T, which thresholds eye, head, and gaze (eye+head) speed (Agtzidis et al., 2019). The original thresholding system was simplified to classify the following types of eye movements: saccades (a high-speed ballistic eye movement), fixation (a period of low to no eye speed), smooth pursuit (a period where the eyes are moving to foveate a moving stimulus), VOR (a period of low to no gaze speed but the eyes are moving in the head to compensate for head motion), and head pursuit (a period of low to no eye speed but gaze is moving, driven by head motion in order to foveate a moving stimuli). A secondary form of smooth pursuit was also classified as smooth pursuit with compensatory VOR (a period where gaze is moving to foveate a moving target and the eyes are moving relative to the head to compensate for head motion). This classification is tested during temporal epochs when smooth pursuit eye movements are likely (i.e., when subjects must track a moving stimulus) and compared to other epochs when smooth pursuit is unlikely (i.e., when the eyes must foveate a static object). As opposed to previous work that has used eye tracking with HMDs, presenting more complex or naturalistic scenes (e.g., 360° videos; Rai et al., 2017; Haskins et al., 2020; Kim et al., 2020), here, stimulus presentation is strictly controlled while viewing behavior (i.e., the movement of the eyes and head) is not. The use of simplified stimuli, similar to those used in previous, 2D display paradigms, allows for an easier comparison to previous results in order to explore how head movements may interact with the execution of eye movements or underlying cognitive processes. The paradigm presented here is generated using a graphical user interface (GUI) specifically designed to allow future researchers to adapt stimulus parameters such as eccentricity and motion speed, in the continued effort to understand how well-studied eye movement phenomena may or may not change when subjects’ viewing is less restricted. The strict control of stimulus presentation is meant to elicit predictable eye movements such as saccades and smooth pursuit in the presence of head motion. This, coupled with the ground truth knowledge of the location of stimuli relative to the viewer’s gaze direction, makes this dataset uniquely beneficial to the development of more automated eye movement classification algorithms.

## Materials and Methods

## Ethics Statement

This experiment was approved by the Institutional Review Board at the United States Air Force Academy (USAFA) and United States Army Research Laboratory (ARL) under Project Number ARL 19–122. All procedures were in accordance with the Declaration of Helsinki.

## Subjects

Twenty-four subjects (United States Air Force Academy cadets; nine female, average age 19.3years) were tested and received course credit for their participation. Subjects were recruited through Sona Systems and provided written informed consent prior to experimentation. All subjects had normal or corrected to normal vision.

## Apparatus

Experimental procedures were designed using the Unity gaming engine.^1^ Stimuli were presented to an HTC Vive VR headset (1,080×1,200 pixels per eye, 90Hz refresh rate, 110° field of view) with integrated eye tracking from Tobii Technologies (120Hz sampling rate, Tobii Pro SDK) using a Corsair One PC (Windows 10, Intel Core i9 CPU @ 3.6GHz, 64-bit, Nvidia GeForce RTX 2080Ti, 32GB RAM) and two external lighthouses used for tracking head position. Subjects were given instructions and practiced to correctly position the VR headset prior to experimentation. Subjects were comfortably seated in a fixed position chair. The Tobii Vive was used here as it is a fully integrated system with an estimated accuracy of 0.5°. Other systems such as the Pupil Labs eye tracker can be added to HMD systems and offer higher tracking frequency (200Hz); however, there is slightly poorer tracking accuracy tracking (1.0°).

Lab Streaming Layer (LSL; available here: https://github.com/labstreaminglayer/LSL4Unity) was used to synchronize eye and head tracking data with button responses and stimulus presentation. LSL is a network-based recording software designed to integrate multiple data streams with sub millisecond precision (Kothe, 2014).

## Calibration

The standard five-point calibration contained in the Tobii Pro SDK was implemented before each block of trials. Calibration points were sequentially presented, one each at the four corners of an imaginary square and the middle point centered on the subjects forward gaze position (in Unity meters: corner points +/−0.3x, +/−0.15y, 1.2z, middle point 0, 0, 1.2). Subjects fixated the center of each point, which started at 0.1m (4.77 degrees of visual angle, dva) in diameter, and shrunk down over the course of fixation until it became invisible, indicating a successfully registered calibration point. Each of the four calibration points was positioned 15.62dva relative to the middle point. All points were presented in an orthogonal plane at a fixed distance of 1.2m. If fixation was interrupted during calibration or the calibration point did not disappear, calibration was restarted. While, we did not record the number of calibration attempts or subsequent validation of the calibration, trials did not start until a successful calibration (i.e., all five points were fixated and registered by the software as completed) was accomplished. No subjects were removed due to poor calibration.

## Units of Measurement in Virtual Environments

Unity objects (stimuli) are defined in world coordinate using notional or approximate meters. However, a more precise and useful metric for vision scientists is dva. As such, both are reported in this paper. It is important to note that the degrees of visual angle are approximate. The screen inside the headset does not fully cover the natural human field of view, leading to a small binocular effect. The fields of view of the virtual cameras are manipulated to counter this effect to make using the headset more comfortable, at the cost of some slight size distortion.

## Graphical User Interface For Paradigm Creation

**Figure 1:**
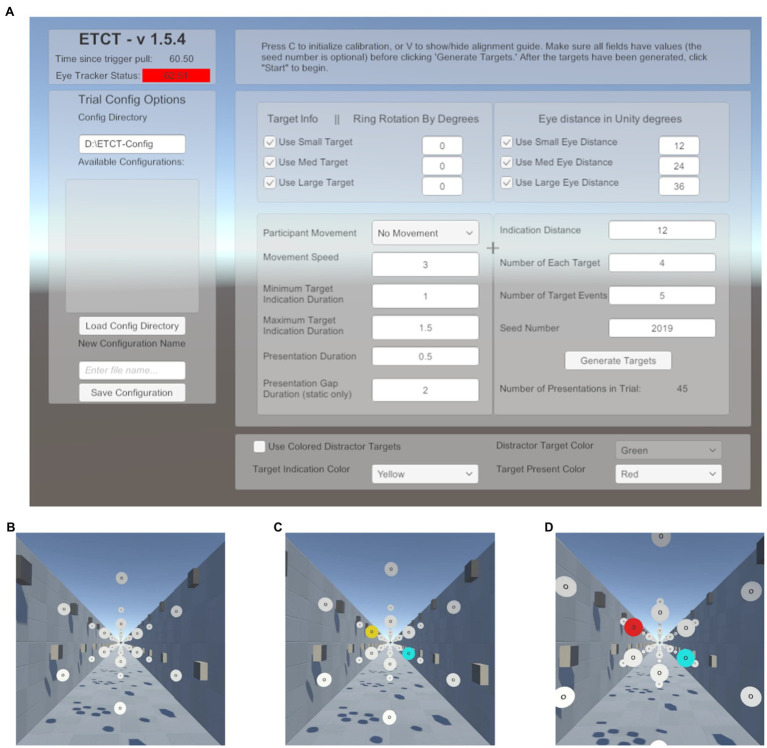
Paradigm schematic. **(A)** Graphical user interface (GUI) presented at the start of the experiment. Here, the researcher can input a number of parameters to alter stimulus presentation. This list includes target parameters such as size as well as the motion (or lack thereof) of the participant through the environment. A list of available parameters and the values set in the current experiment is included in the Supplementary Materials. **(B)** Start of a trial. Subjects foveate the recentering cross ahead of them. **(C)** Target indication phase. Subjects are told to saccade to the yellow disk in preparation for the target and ignore the cyan disk. **(D)** Target presentation. Subjects must maintain fixation on the yellow disk until the target is presented, at which time the yellow disk turns red and the “O” at the center of the disk is replaced with a “C” facing either the left or the right. Subjects report the direction of the “C” using the virtual reality (VR) controls and return their gaze to the recentering cross to await the next trial.

To make this paradigm adaptable for future research, the GUI was included in the software development (Figure 1A). Researchers using the supplied code can leverage a GUI to change multiple parameters related to target size, target position, movement speed, quantity, randomization, trial numbers, and temporal contingencies.^2^ For example, researchers can set the size and rotation of targets. Likewise, researchers can change the perceived motion from an observer moving through a space of static disks to the disks moving past a static observer by changing whether the background moves with the participant or with the disks. Additionally, the GUI provides a number of status checks such as indicators that the eye tracker and hand controllers are connected. The intention behind the creation of this GUI was to lower the bar of entry for future researchers and provide a highly flexible generator of visual search or discrimination tasks. The parameters, cited below, were those used in the data reported here. Additionally, all parameters available in the GUI as well as a list of recorded data can be found in the Supplementary Materials.

## Visual Parameters and Trial Structure

In the virtual environment, a directional light (RGB: 1, 0.95, and 0.84) was used, rotated 50° in the x-axis and −30° in the y-axis in the Unity coordinate system. This had the effect of lighting the scene from over the subject’s right shoulder, directed toward their left foot. This ensured all target surfaces were lit. Furthermore, the light created shadows from surrounding objects to provide a sense of depth and create a realistic perception of motion in Dynamic trials (see details below).

Subjects performed 288 trials of a cued, two-alternative forced choice, target discrimination task. On each trial, subjects were instructed to foveate a recentering cross (0.18m; 0.94dva) placed 11m in front of them. The recentering cross was surrounded by an array of white disks (RGB: 1,1,1), each 1m in diameter evenly spaced on an imaginary circle (Figure 1B). Each of the surrounding white disks had the letter “O” at its center (RGB: 0,0,0). On each trial, one of the white disks was cued by turning yellow (RGB: 1, 0.92, and 0.016). Upon the appearance of the cue, subjects were instructed to make a saccade to the “O” in the center of the yellow disk as quickly as possible and foveate the center until a subsequent target was presented. The presentation of the cue was not gaze-contingent meaning it would occur even if the subject’s eyes were not on the recentering cross. Simultaneously with the yellow cue presentation, a counterpart disk, at a diametrically opposite point along the ring relative to the cued disk, turned cyan (RGB: 0,1,1; Figure 1C). The cyan distractor disk was included for the purposes of piloting for a follow-up study using EEG in order to control sensory input between visual fields and prevent reflexive saccades. Our analyses focus only on the target disk and thus the counterpart cyan disk is not discussed further in this paper. After 600–1,600ms the yellow disk turned red (RGB: 1,0,0) and simultaneously the “O” label was replaced with the target, a “C,” faced either to the left or right (Figure 1D). The interval between cue (yellow disk) and target (red with “C”) was used to allow subjects enough time to locate and saccade to the disk as well as provide variable of tracking the disk, relative to target onset. Subjects were told to use the VR controllers, one in each hand, to report if the “C” was open to the left or the right, an equally probable occurrence that required responses from the left and right controllers, respectively. The “C” was present for 1,000ms. Subjects were instructed to return their gaze to the recentering cross as soon as a response was given. There was an average total of 6.4s between the start of one trial (the onset of the cue) and the next. Responses were counted as valid if they occurred between the presentation of the target and the onset of the cue in the next trial. Only first responses were analyzed.

Subjects experienced both Static and Dynamic trials. In Dynamic trials, subjects “moved” through the environment at 5m/s. This movement was strictly in the virtual environment as subjects remained stationary in their chair throughout the experiment. The perception of motion was induced by controlling the lighting/shadows in the environment and moving the point of view camera through space. Dynamic trials were included in order to elicit smooth pursuit eye movements. The speed was chosen as a balance between wanting the eyes to move quickly enough to elicit smooth pursuit but not so fast that there was not ample tracking time before a cued disk passed out of view of the participant.

Disks were always cued (turned yellow) 32m from the subject in the Dynamic trials. In the Static condition trials, the disks were stationary and were cued either 13 or 32m from subjects. This translated to the disk being approximately 4.4dva when cued at its closest (13m) location and 1.8dva at the farthest (32m), relative to the subject. Consequently, the “O” (and subsequent “C”) on the disks were then 1.89dva at 13m from participants and 0.77dva at 32ms from participants. The two cueing distances were used to make Static trials more comparable to Dynamic where the cue traveled closer to the subject throughout the trial. Disks were cued in the periphery (20dva from the recentering cross) and the parafovea (6dva from the recentering cross) in both Dynamic and Static trials. Target disks and cue durations were randomly selected using a random seed generator. All subjects performed the task using the same seed. That is, each subject experienced the same random order. Subjects performed two blocks of 48 trials in the Static condition, where the cued disk was 13m from the subject, the Static condition where the cued disk was 32m from the subject, and the Dynamic condition. The block order was counterbalanced. Calibration was performed prior to each block. Data from each participant can be found online at https://osf.io/p8g94/.

## Simulator Sickness Questionnaire

A concern in VR with HMD experimentation is inducing simulator sickness in subjects due to the discrepancy between task-induced motion in the virtual environments and the lack of motion in the real environment. Simulation sickness was measured using the Simulator Sickness Questionnaire (SSQ; Kennedy et al., 1993) before and after the experiment. While the SSQ was designed to measure simulator sickness in flight simulators many researchers have adopted its use in VR environments (see Saredakis et al., 2020, for a review). Subjects rated 16 symptoms on a four-point scale (0–3), which were factored into three categories (Oculomotor, Disorientation, and Nausea) and computed into a Total score.

## Eye Movement Classification and Validation

Tobii interpolates eye position coordinates for dropped samples (e.g., blinks or missing eye image) but does not interpolate pupil recordings. Therefore, valid eye position values were defined as timepoints, which had corresponding valid pupil samples. Only valid eye position samples were included in analysis. Blinks were not explicitly defined other than as dropped or invalid samples. All invalid epochs, as well as 40ms before and after the invalid epoch, were considered noise (i.e., invalid) and excluded from classification.

**Figure 2:**
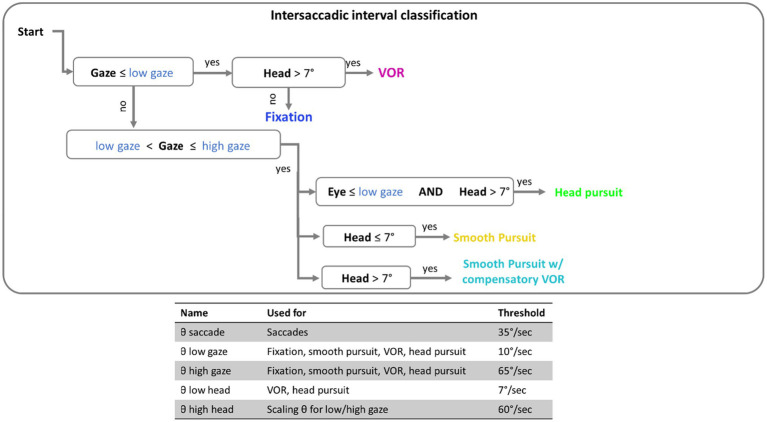
Flow chart of the threshold classification. Intersaccadic intervals were divided into 100ms windows for classification. Threshold in blue font are thresholds that are scaled by the current speed of the head. The table at the bottom contains the complete set of threshold values used.

Here, the term eye speed refers to the angular velocity of the eye, relative to the head. Gaze speed refers to angular velocity of foveation relative to the world (the combined eye and head speed). Before classifications of eye movements were made, a five-sample (40ms) median filter was applied to smooth both eye and head speed data (Engbert and Kiegl, 2003; Engbert and Mergenthaler, 2006; Dimigen et al., 2011). Eye movements were classified by applying a dynamic threshold to gaze and eye speed that is scaled by the current head speed: threshold_scaled_=(1+*v_head_*/60)*threshold, where *v_head_* is the velocity of the head at a given time point (see Agtzidis et al., 2019, for details). Saccade detection was performed first. The label “saccade” was applied to all time points in windows of 20ms or longer, where eye speed exceeded the scaled velocity threshold [for saccades this would be (1+*v_head_*/60)*θ_Saccade_, where θ_Saccade_ is the saccade threshold when the head is stationary, 35deg/s; see Figure 2]. For analysis, only saccades over 3° in amplitude with a peak velocity under 1,000 deg./s were included. This velocity cutoff was based on previous research (Holmqvist et al., 2011; Ries et al., 2018) to exclude improbable eye movements, and 3° was used to exclude small eye movements around the recentering cross.

Intersaccadic intervals were classified in 100ms epochs based on a set of thresholds (see Figure 2) for the gaze speed and head speed. The implementation of thresholding here has been outlined in the flow chart of Figure 2. If gaze speed was below the scaled low gaze threshold [that would be calculated as (1+*v_head_*/60)*θ_lowgaze_, where θ_lowgaze_ is the lower bound gaze threshold when the head is stationary, 10deg./s] then the window was assigned a label of “VOR” or “fixation” depending on if the head above threshold (7deg./s). If gaze was moving (above scaled low threshold), the epoch was classified as a “head pursuit” if the eye speed was below threshold, “smooth pursuit” if the head speed was below threshold, or “smooth pursuit with compensatory VOR” if both head and eye speed were above threshold.

The classification algorithm was compared against ray-casts of each gaze sample. Ray-casting is when an imaginary ray is generated based on the instantaneously estimated gaze vector and projected until it collides with an object in the virtual environment. This offers an estimation of what stimulus the eyes were foveating at a given time (Watson et al., 2019). Due to the experimental control of VR, location and speed information of each stimulus is known. Combined, this information can be used to identify epochs, where the participant was foveating on either a particular moving or stationary stimulus. To quantify the classification accuracy of our data, we computed the percentage of time points when the eye was foveating a stationary object (the recentering cross or static target) that were erroneously classified as smooth pursuit (including smooth pursuit with compensatory VOR) or head pursuit. This was compared to the percentage of pursuit labeled time points when the eyes were foveating moving objects (i.e., targets in the Dynamic condition). VOR was more difficult to test for as, unlike pursuit, no part of the paradigm necessarily demanded the subject engage in VOR to complete the task. For exploratory purposes, we compared situations when VOR may have been more likely (i.e., just after a saccade to a peripheral static target disk, where the eyes would be left at a more extreme angle and therefore encourage head rotation while maintain gaze on a fixed point) to situations, where VOR may have been less likely (i.e., just after a saccade to a parafoveal static target disk, where perhaps head rotation is less necessary to ensure comfortable gaze position, or during a fixation on the recentering cross).

## Results

### Simulator Sickness

No subjects experienced symptoms severe enough to withdraw voluntarily from the study. SSQ scores were evaluated using a 2×3 repeated measures ANOVA with time (Pre, Post) and category (Oculomotor, Disorientation, and Nausea) as factors. Greenhouse-Geisser values are reported for the interaction between time and category, which violated sphericity assumptions (Mauchly’s *W*=0.741, *p*=0.037). Sidak corrections were used for multiple comparisons adjustment. There was a main effect for time *F*(1,23)=14.67, *p*=0.001, *η*^2^=0.39 indicating higher average ratings post experiment (12.9, SE 2.5) compared with the start (3.89, SE 1.2) of the experiment. There was a significant main effect for category, *F*(2,46)=11.99, *p*<0.001, *η*^2^=0.34, with Oculomotor (12.95, SE 2.4) ratings higher than Disorientation (6.67, SE 1.7) and Nausea (5.57, SE 1.2); both *p*<0.01. The time by category interaction was also significant *F*(2,46)=5.76, *p*=0.01, *η*^2^=0.2 indicating larger post-pre differences in the Oculomotor with respect to the other two categories. An additional paired-samples *t*-test examined the pre/post difference for the Total scores with significantly higher Total scores at the end (15.9, SE 3) compared to the beginning (4.83, SE 1.5) of the experiment *t*(23)=3.8, *p*=0.001.

It should be noted that while simulator sickness increased from the start of the experiment to the end, there was no significant difference between saccadic reaction times, *F*(1,23)=0.5, *p*=0.45, or button press reaction times, *F*(1,23)=2, *p*=0.16, between the first and last block of the experiment indicating simulator sickness did not have a significant effect on performance.

### Eye and Head Responses to Targets at Parafovea and Peripheral Eccentricities

On average, 94% (*SD*=4%) of eye data samples were valid in each subject’s datafile. This high percentage of valid data can be attributed in part to the eye tracker cameras being embedded inside the headset. This prevents the head and eyes from moving outside the tracking box causing dropped samples, which can occurs in monitor tracking systems when the eyes move outside the confines of the eye tracker (Hessels et al., 2015). This is a feature shared across mobile eye tracking systems more generally (e.g., ETGs and augmented reality devices).

**Figure 3:**
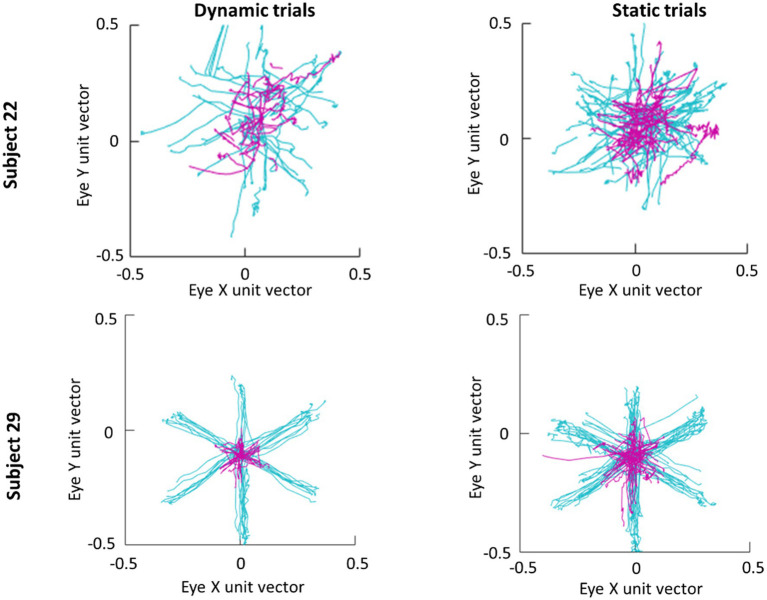
Scan paths of first saccade made in each trial for subjects 22 (top row) and 29 (bottom row). Trials where the cue appeared in the parafovea and periphery are plotted in magenta and cyan, respectively.

**Figure 4:**
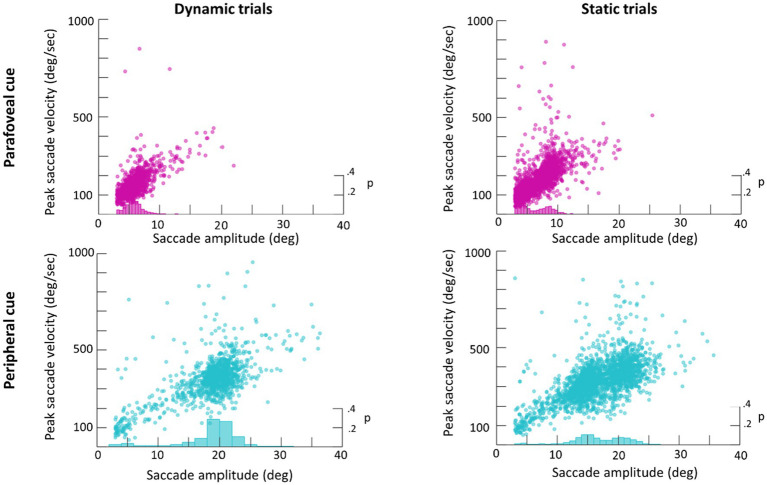
Main sequence scatter plot of saccade amplitude vs. peak amplitude for Dynamic (left) and Static (right) trials. Frequency histograms are plotted on the right y-axis.

An average of 365 detected saccades (*SE*=28) were excluded from each participant’s data for having an amplitude under 3° and an average of 28 detected saccades (*SE*=2) were excluded for having a peak velocity over 1,000° deg./s. In total this averaged to 15% of detected saccades being excluded from analysis. Scan paths for the first saccade in each trial are plotted in Figure 3 as an example of a low (subject 29) and high (subject 22) scan path variability. During the Dynamic condition an average of 1.36 saccades (*SE*=0.06) were made in parafovea (6° of visual angle from the recentering cross) trials and 1.60 saccades (*SE*=0.09) in periphery (20° of visual angle from the recentering cross) trials. An average of 1.18 (*SE*=0.04) saccades were made in parafovea trials and 1.32 (*SE*=0.06) saccades in periphery trials during the Static condition. The main sequence shown in Figure 3 exhibits the saccade amplitude by peak velocity relationship for the first saccade in each trial separated by parafoveal and peripheral trials. The mean amplitude of saccades to peripheral cue locations was 17° (*SE*=0.25) and 18° (*SE*=0.39) in Static and Dynamic trials, respectively (Figure 4). For trials, where the cue appeared in the parafovea the mean saccade amplitude was 6° (*SE*=0.15) and 6° (*SE*=0.17) in Static and Dynamic trials, respectively. The skewness and kurtosis were also calculated for Dynamic parafoveal trials (*γ*=2.53, *k*=13.50), Dynamic peripheral trials (*γ*=−0.89, *k*=5.45), Static parafoveal trials (*γ*=1.06, *k*=5.51), and Static peripheral trials (*γ*=−0.15, *k*=4.01).

**Figure 5:**
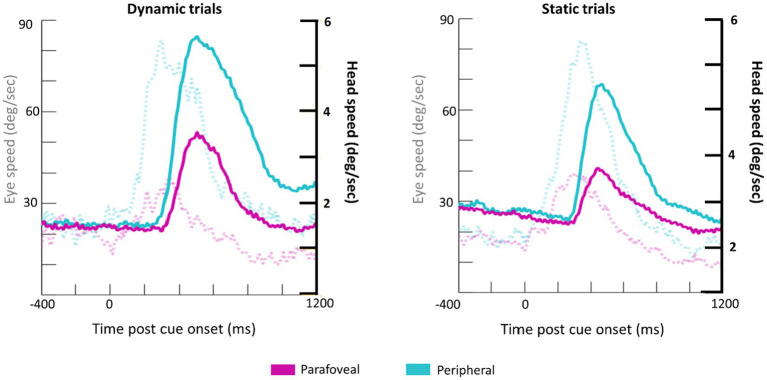
Grand average waveforms for eye speed (transparent, dotted lines; y-axis on the left) and head rotational speed (bold lines; y-axis on the right) for Dynamic (left) and Static (right) trials.

Subjects made a combination of eye and head movements to shift their gaze to the cued target disk (Figure 5). Unsurprisingly, larger and faster eye and head movements were made when the cue appeared in the periphery. The time course of head and eye speed shows that, on average, head rotational speed peaks about 200ms after peak saccadic movement. Together, the head and eye movement measurements in response to the cue onset serve as a quality control check of the eye tracking data collected from HMDs.

### Eye Movement Classification

**Figure 6:**
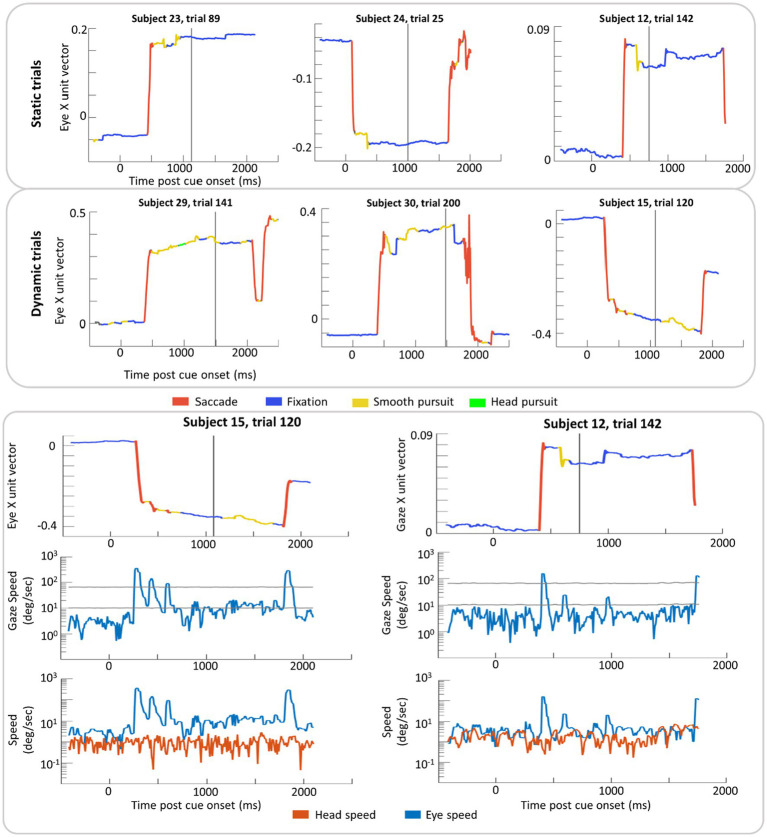
Example trial plots of the eye unit vector along the x-axis for Static and Dynamic trials (top section). In the bottom section, two trials from the top (subject 15, trial 120 and subject 12, trial 142) have been expanded to include the log linear transform of gaze speed plotted with scaled high and low thresholds (grey lines) in addition to the log linear transform of eye and head speed (bottom row). These transformations were done in order to plot instances of high speed without losing detail in slower speed time periods. In each graph, time zero marks the time of the cue (yellow disk) onset and the grey vertical line indicates the time the target was presented. Red lines indicate time windows labeled saccade and blue lines indicate time windows labeled fixation. Yellow and green lines indicate windows in of smooth pursuit and head pursuit, respectively.

Example trials with classification labels are plotted over eye position in Figure 6. VOR classification was rare in this dataset (2% of time points on average) with 1% for parafoveal targets and 3% for peripheral targets. When the ray-cast gaze vector was on the recentering cross, 10% of time points were classified as smooth pursuit in both Static and Dynamic trials. When the gaze vector was on the target, 11% of time points were classified as smooth pursuit in the Static trials compared to 19% in Dynamic trials (where smooth pursuit is likely to occur).

**Figure 7:**
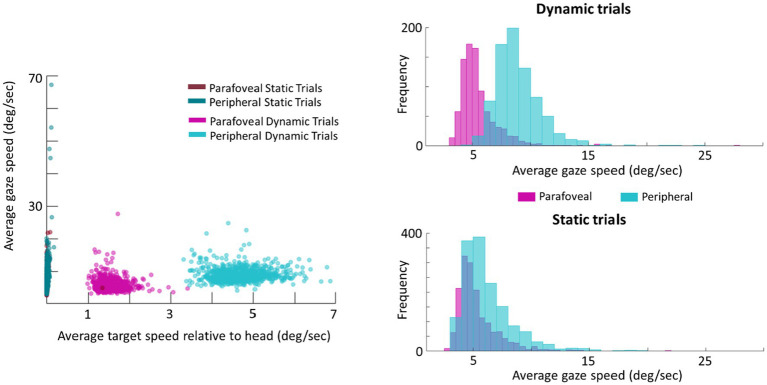
Scatter plot of average gaze speed compared to average target speed (left). Histogram of gaze speed for Dynamic trials (right, top) and Static trials (right, bottom).

**Figure 8:**
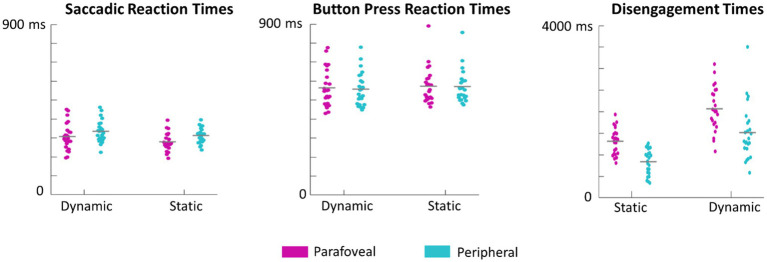
Swarm plots of subject averages in saccadic reaction times, button press reaction times, and disengagement times. It is important to note that saccadic reaction times are calculated as the difference between the time of saccade onset and the time of the cue onset. Button press reaction times are calculated as the difference between when the button was pressed and the time of target onset. Disengagement time is the difference between the time the eyes first left the target and the time of target onset.

As a follow-up analysis, the average gaze speed was calculated for the longest intersaccadic interval, where the mode ray-cast label of time samples was the target (this was done as some trials contained multiple intersaccadic intervals, where the eyes were foveating on the target). To limit the influence of potential smaller or catch-up saccades on the average gaze speed, time points in which the eye speed exceeded 20deg./s were excluded from this calculation. This average gaze speed was then plotted against the target’s speed relative to the head (Figure 7). Plotting the distribution of gaze speeds for each condition, it is apparent that, with a lower gaze threshold, smooth pursuit classification may improve for Dynamic trials in which the target disk was cued in the periphery as the average gaze speed was 9.1deg./s (*SE*=0.2deg./s, *Median*=9.1deg./s). However, Dynamic trials in which the target disk was cued in the parafovea elicit a gaze speed (*M*=5.7deg./s, *SE*=0.3deg./s, *Median*=5.3deg./s) that is difficult to isolate from Static parafovea trials (*M*=5.5deg./s, *SE*=0.2deg./s, *Median*=5.2deg./s) and Static peripheral trials (*M*=6.6deg./s, *SE*=0.3deg./s, *Median*=6.4deg./s). Repeating the validation procedure outlined above and separating peripheral and parafoveal targets in the Dynamic conditions shows a smooth pursuit classification of 33 and 10%, respectively (as a reminder 11% of time points were classified as smooth pursuit for static targets). This suggests that the target speed (and associated gaze speed) in Dynamic parafoveal trials was too slow to classify as smooth pursuit using the thresholds in the classification algorithm (Figure 8).

### Effects of Target Eccentricity and Motion on Task Performance

Saccade and button reaction time to the cue and target, respectively, were analyzed to explore whether target motion or eccentricity affected the speed of subject responses. There was no significant main effect of motion, *F*(1,23)=1.8, *p*=0.19, eccentricity, *F*(1,23)=1.7, *p*=0.20, or interaction between motion and eccentricity, *F*(1,69)=1.2, *p*=0.3, on button press response times. However, first saccades (as defined by the first saccade made after the onset of the target disk cue that measured over 3° in amplitude) were initiated earlier when the target was in the parafovea compared to the periphery, *F*(1,23)=27, *p*<0.001 (Figure 7). There was no significant effect of motion on saccadic reaction time, *F*(1,23)=3.9, *p*=0.06 or interaction between motion and eccentricity, *F*(1,69)=0.7, *p*=0.4. We also performed a more conservative analysis, where only trials in which the eyes successfully executed a saccade that went from the recentering cross to the target disk were included. This criterion limited the number of valid trials as subjects often made multiple saccades from the recentering cross to the target disk. As such, 14 subjects had at least 100 trials meeting the criterion and were included in this secondary analysis which also found a significant effect of eccentricity on saccadic reaction times, *F*(1,13)=41, *p*<0.001, but not of motion, *F*(1,13)=2.8, *p*=0.11, nor any interaction, *F*(1,13)=1.9, *p*=0.19. For button reaction times, there was not significant main effects or interactions with the more conservative criterion.

Subjects were instructed to return their gaze back to the recentering cross immediately after button response. This disengagement time, defined as the time between target presentation and the time at which the eyes left the target, was also evaluated. Both eccentricity, *F*(1,23)=87, *p*<0.001, and motion, *F*(1,23)=69, *p*<0.001 significantly influenced the disengagement time with the eyes leaving dynamic targets and those cued in the periphery earlier (Figure 8). There was no significant interaction between motion and eccentricity, *F*(1.69)=0.55, *p*=0.5.

## Discussion

A fundamental goal of vision researchers is to understand how the human visual system operates in the natural environment. While requirements for experimental control and technological limitations may have necessitated the use of simplified stimuli presented on 2D monitors, the aim has always been to use these results to elucidate mechanisms of real-world vision. While these previous findings have provided an important foundational knowledge, it is essential to ensure that effects seen in the laboratory do in fact translate to the outside world and examine cases in which they do not. For instance, subjects do not exhibit the same detriment in recognition of a scene from a new viewpoint when they themselves have moved to the new viewpoint compared to when the scene is presented in rotated form (as is typical in 2D display experiments; Simons and Wang, 1998). Such findings demonstrate that there are components of natural vision, such as body movement, that are fundamentally integrated with cognition, but are often missed in classic monitor-based experiments. With this in mind, we used a VR HMD with eye tracking technology to study dynamic eye and head movement patterns within a visual discrimination paradigm to induce naturalistic gaze patterns within an immersive yet controlled environment.

The purpose of this work was to demonstrate the capabilities and possible applications of this system as well as to encourage future researchers to incorporate head movements in their exploration of the visual system. To that end, we provided the GUI to lower the bar of entry for vision researchers new to developing paradigms within VR. This GUI allows researchers to quickly and easily set-up a variety paradigms, altering characteristics of the stimuli as well as the relative motion between the participant and the stimulus of background. The intent here is to provide a jumping off point for researchers that may want to move in to the VR with HMD space but hesitate at the upfront programming cost.

The current work used a previously published threshold algorithm designed for use in 360deg. viewing environments to classify various eye movements such as saccades, fixations, smooth pursuit, and VOR (Agtzidis et al., 2019). To test smooth pursuit classification the current work used the ground truth data about the position of virtual objects to estimate time periods, in which the gaze should be stationary (i.e., when foveating on static objects) compared to when gaze should be engaged in smooth pursuit (i.e., when foveating on moving objects). Separating parafoveal vs. peripheral trials showed that one of the potential contributors to the poor classification accuracy of smooth pursuit was the fact that the relative movement of the disks was too slow to elicit the minimum gaze speed necessary to achieve a “moving” gaze designation. Smooth pursuit classification improved in Dynamic trials, where the target disk was cued in the periphery because targets in the periphery have a higher angular velocity relative to the head. Conversely, in Dynamic trials where the target was cued in the parafovea, tracking the target did not elicit gaze speeds that were faster than those of static targets (see an example trial video at https://osf.io/p8g94/). It should be noted that these thresholds were originally set based off an annotated dataset collected by Agtzidis et al. (2016) and therefore could require adjustments based on the speed characteristics of the current stimuli. Smooth pursuit is generally difficult to classify without information about the environment (Agtzidis et al., 2016); however, ideally a classification system does not need to be tailored the specific dataset. Using the directional change of gaze may improve smooth pursuit classification as presumably there would be more coherence in the direction of eye movements when the eyes are tracking a moving stimulus compared to when they are moving around while foveating a static stimulus.

Of course, a limitation of our approach is that it only examines time points in which the eyes are foveating a moving object to test for smooth pursuit, while smooth pursuit can occur without the target object being in the fovea, often necessitating catch-up saccades (de Brouwer et al., 2002). However, using the ray-cast data to isolate when the eyes foveate the target, while not the most conservative approach, should provide a measure of ground truth. It should be noted that on trials where the target was moving faster (Dyanmic, peripheral trials) the accuracy rate (33%) was comparable to rates found in the original thresholding paper (29–38%; Agtzidis et al., 2019). The results of this technique indicate that strict thresholding is not always sufficient for detecting when the eyes are tracking a moving target at slower speeds. Together, our results demonstrate the complementary benefit of having the ground truth knowledge of stimulus trajectories in VR to similar datasets which use 360° video (David et al., 2018).

### Considerations and Limitations of Building Experiments Using HMDs With VR

While there are a number of advantages in using HMD with VR to explore visual processes there are also limitations to consider. For example, given the results obtained using the SSQ, VR researchers should consider ways to mitigate simulation sickness such as smaller fields of view and higher frame rates (e.g., Draper et al., 2001; Keshavarz and Hecht, 2011). Simulator sickness is of special consideration when setting up paradigms meant to elicit specific eye movements. For instance, a potential takeaway from the current findings is that faster moving stimuli should be used in order to reliably classify smooth pursuit. However, this may enhance feelings of simulator sickness and require more frequent breaks or other remedial measures.

Another consideration is that the computer running the game engine will experience fluctuating load levels while rendering the environment. This can be due to visual complexity (e.g., dynamic lighting) or gameplay (e.g., simulation of physical interactor) or other background processes. During periods of low load, the computer can render the environment at sufficiently high frame rates (>100 frames per second). However, as complexity increases, the frame rate can drop substantially depending on the processing capacity of the computer. Importantly, the game engine generates many of the metrics, including position and rotation measures, during each frame refresh. Therefore, the majority of data streams can have a variable sampling rate, not under the direct control of the experimenter, and great care needs to be taken to optimize the simulations performance. It is advisable to set a target frame rate that the simulation will not fall below and to use a computer that is powerful enough to maintain a stable rate. If the environment is not optimized appropriately, rendering can still drop below this target frame rate.

While VR platforms allow for more natural movement, they are restricted in large-scale movement, requiring subjects to teleport themselves through larger virtual environments or incorporate a treadmill. Mobile eye tracking systems avoid this limitation, allowing subjects to navigate the world as they normally would. Another benefit to mobile eye tracking systems is that objects in the environment have real, not simulated, depth, potentially resulting in more natural vergence and accommodation responses.

The VR HMD used here offers eye tracking with a 120Hz sampling rate. This the low- to mid-range of sampling frequencies necessary to detect and classify eye movements (Holmqvist et al., 2011). The lower sampling rate of the eye trackers in VR may result in less accuracy for measuring small saccades (e.g., microsaccades) and their associated peak velocities. Additionally, lower sampling rates may impair the ability to effectively use certain gaze-contingent interaction, thus preventing adequate online stimulus display changes. Sampling rate of in HMD eye trackers should then be considered not only when designing paradigms but also when comparing results to other eye tracking systems that may have higher sampling rates available.

Another limitation that should be considered is in relating ray-casting to perception. Ray-casting can be a helpful way of labeling an object within foveal vision during a fixation. However, due to noise in the gaze vector estimation and to decreased accuracy compared to desktop eye trackers, it is difficult to accurately classify what object is being foveated if objects in the environment are too close to one another. Watson et al. (2019) offers an alternative to this approach with a “shotgun” ray-cast that returns a list of objects contained in the area surrounding gaze position. However importantly, neither a pin-point or shotgun ray-cast gives explicit insight into what objects in the visual field are actually attended to or encoded into memory and this should be kept in mind when drawing conclusions of perception from ray-cast data.

Lastly, the HMD used here utilizes “Outside In” tracking, requiring external lighthouses containing infrared scanners to be mounted in opposing corners of the tracking area. These lighthouses contain spinning mirrors, and so are susceptible to vibrations if not firmly mounted. Also, reflective surfaces could potentially disrupt the headset’s ability to track the lighthouses. It is important to reduce reflective surfaces and firmly mount lighthouses when using a headset with Outside In tracking. Additionally, newer versions of this technology, such as the Tobii Vive Pro, allow for the installation of two additional lighthouses (which is the maximum number available with the system used here) which may help to address tracking issues (Niehorster et al., 2017).

## Conclusion

Virtual reality used in conjunction with HMD offers a potential solution to vision researchers by offering a balance between allowing more naturalistic behavior in participants without sacrificing strict experimental control. Here, we offer a demonstration of some of the capabilities of this system as well as the GUI for future researchers to be able to quickly launch a variety of visual search paradigms to suit research needs. There are a number of technological hurdles to consider when experimenting within VR which we have outlined above. However, overall this technology offers a promising space for understanding how vision is performed in the natural environment.

## Data Availability Statement

The raw data supporting the conclusions of this article will be made available by the authors, without undue reservation.

## Ethics Statement

The studies involving human participants were reviewed and approved by ARL Human Research Protection Program. The patients/participants provided their written informed consent to participate in this study.

## Author Contributions

CB developed all Unity code for the paradigm and its GUI as well as collected data. AR came up with the idea and designed the experimental task and collected data. CC-F performed the analysis and wrote the manuscript. JT provided support for the creation of the paradigm as well as subsequent data analysis. All authors contributed to the article and approved the submitted version.

## Conflict of Interest

The authors declare that the research was conducted in the absence of any commercial or financial relationships that could be construed as a potential conflict of interest.

## Publisher’s Note

All claims expressed in this article are solely those of the authors and do not necessarily represent those of their affiliated organizations, or those of the publisher, the editors and the reviewers. Any product that may be evaluated in this article, or claim that may be made by its manufacturer, is not guaranteed or endorsed by the publisher.

## Footnotes

^1^U. Technologies. Unity – game engine. http://unity3d.com/

^2^ https://osf.io/p8g94/
