# Supplementary material for: A Case for Studying Naturalistic Eye and Head Movements in Virtual Environments
Source: Front Psychol. 2021 Dec 31;12:650693. doi: 10.3389/fpsyg.2021.650693 (PMC8759101; doi:10.3389/fpsyg.2021.650693)
Supplement: Supplementary file 1 [file Data_Sheet_1.PDF]

Supplemental Materials

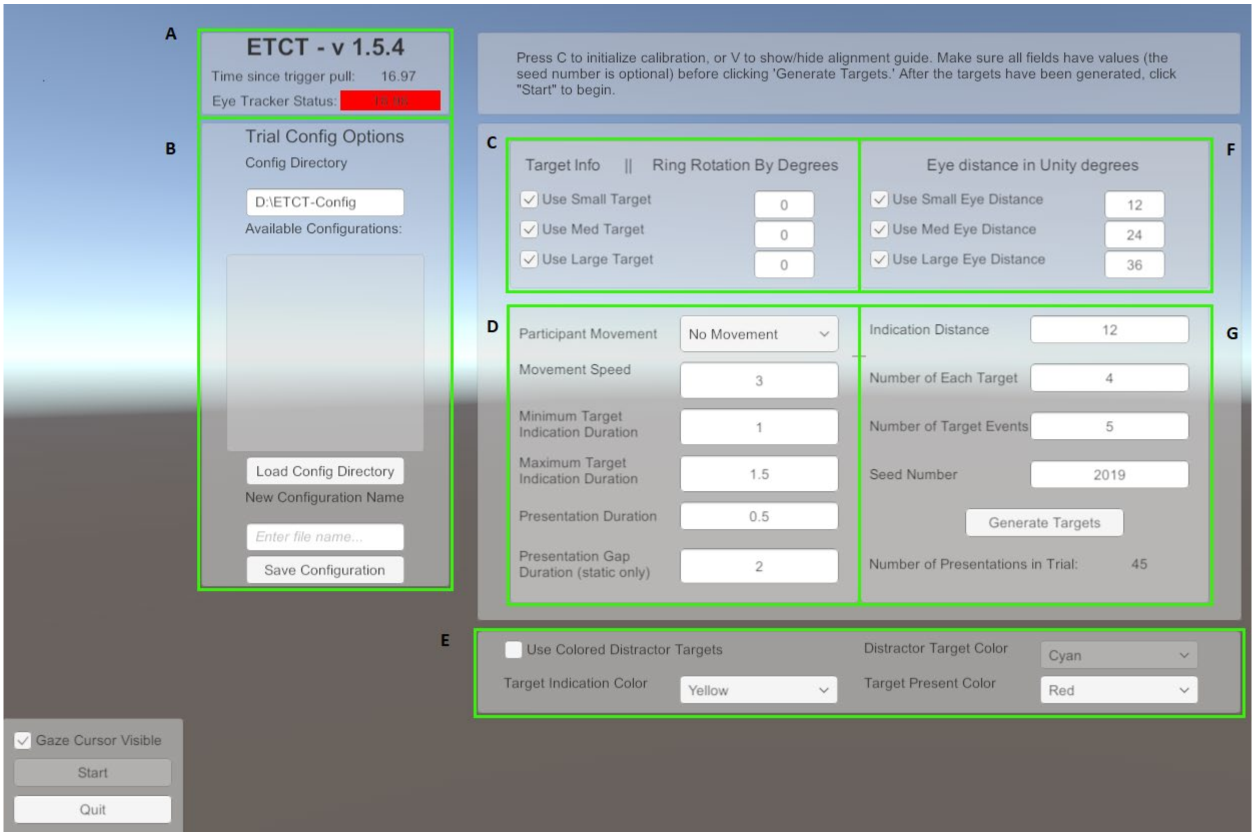

Graphical user interface parameters:

In the bottom left is the Start panel. Changing any of the settings on the rest of the menu causes the start button to become disabled until disks (i.e. potential target locations) have been (re)generated. There is also a toggle for a visible gaze cursor. In the top right is a rolling average of Frames Per Second for the simulation.

A: Status Info
Provides the version number, as well as some quality of life readouts. Time since trigger pull is the time in seconds since the participant last pulled either of the controllers' triggers. Eye tracker status displays green if the eye tracker is connected and the program is receiving data. Otherwise, it displays red, with the time since the last sample was received in seconds.

B: Config Options
After specifying a directory, particular configurations of program settings (sections C-G) can be saved, for easy recreation later. A file name must be entered into the text box at the bottom of the panel before saving. Once configurations have been saved, clicking on the "Load Config Directory" button loads any config files found at the path specified in the config directory text

box. Clicking on one of the loaded config files causes the settings in sections C-G to change to match the config file's settings.

C: Target Info Panel 1
This section allows changing which sizes of disks will appear in the trials, as well as how much each "ring" of disks will be rotated by. This does not rotate individual disks.

D: Experiment Settings Panel 1
Participant Movement can have 3 possible values: No movement, where the participant and the disks are both static, Participant Moves, where the participant moves through a series of rings of disks, and Targets move, where rings of disks move past the participant.

Movement speed determines how fast either the player or the disks move in m/s if movement is selected.

Minimum Target Indication Duration is the minimum time in seconds the disk will spend in its indication state (i.e. cued) before transitioning to its presenting (i.e. target) state.

Maximum Target Indication Duration is the maximum of the same metric.

Presentation Duration is how long the target will be presenting, in seconds. This is the point at which the bullseye on the target changes to a C, and the participant must decide which way the C is pointing.

Presentation Gap is how long, in seconds, between the end of one target presenting and the beginning of the next target being indicated, in experiments where there is no movement.

E: Target Presentation Visual Settings
This panel sets how the indicate-present cycle will look. When a disk enters the indicated state, it's color will change from white to the color selected from the drop down for Indication color. When the disk goes from indication to presentation, its color gets set to the present color, and then back to white once the presentation step is finished. Finally, if the "Use Colored Distractor Targets" toggle is checked, then the disk directly opposite to the indicated disk (if there is one) will change to the indicated color, and back to white after the presentation step is finished. This only works with even numbers of disks in a ring, as otherwise there is no "opposite" target to distract with.

F: Target Info Panel 2
This section determines the number of rings (up to 3) and their angle off of the central axis. The angles listed must be in ascending order for the target generation to work properly.

G: Target Generation Panel
The indication distance is how many meters from the participant the disk should be when it changes from inactive to indicated. Note that this is the set distance for all disks regardless of

angle off of the central axis, meaning that each disk on a ring will be the same distance from the participant when one of the disk is indicated.

The number of each disk is how many times in each ring each selected disk size will appear. If that number is 2, and only small disks are selected, each ring has 2 small disks. If that number is 2 but both small and large disks are selected, then each ring will have 2 small and 2 large disks.

Number of target events determines how many times each size disk in each position should be presented. For example, if a ring has 1 small disk and 1 medium disk, and number of disk events is set to 2, then there will be two presentations of the small target when it is at the top of the ring, and two presentations when it is at the bottom of the ring, for four presentations of the small target in total.

The seed number is used to set the random number generator used to generate targets and presentations. Entering the same number gives the same experiment each time.

Once all parameters have been set, click the "Generate Targets" button to create all of the disks, and prepare to run the experiment. Doing so enables the start button after target generation.

The number of presentations text gives the total number of presentations for all targets.

Datafile structure outline:

Eye Tracking Control Task
This task presents participants with a configurable series of targets in VR. The participant must make an eye movement to the target when the target is indicated. After indication, the target will present a bullseye facing either to the left or the right. The participant must then pull the trigger of the vive controller in the matching hand. Finally, the target returns to normal, and the participant returns their gaze to the center marker.

LSL Channel Listing
There are 10 LSL streams created by this experiment:

- TobiiEyeData
- ParticipantActions
- HeadPose
- SessionEvent
- TargetNumericalData
- TargetOtherData
- SessionSettings
- GazeTargetNumericalInfo
- GazeTargetOtherInfo
- GazeData

- AngleDeltas

TobiiEyeData

This is the stream for the raw samples from the Tobii Eye tracker. There are 26 channels, formatted as floating point numbers, as described below.

Channels:
1. Left Pupil Validity: Is the eye tracker effectively tracking the left pupil? 0 if it is not, 1 if it is.
2. Left Pupil Diameter: The diameter of the left pupil in mm.
3. Right Pupil Validity: Is the eye tracker effectively tracking the right pupil? 0 if it is not, 1 if it is.
4. Right Pupil Diameter: The diameter of the right pupil in mm.
5. Left Pupil Position Validity: Is the eye tracker effectively tracking the position of the left pupil? 0 if it is not, 1 if it is.
6. Left Pupil Position X: The position of the left pupil along the X axis
7. Left Pupil Position Y: The position of the left pupil along the Y axis
8. Right Pupil Position Validity: Is the eye tracker effectively tracking the position of the right pupil? 0 if it is not, 1 if it is.
9. Right Pupil Position X: The position of the right pupil along the X axis
10. Right Pupil Position Y: The position of the right pupil along the Y axis
11. Left Gaze Direction Validity: Is the eye tracker effectively tracking the gaze direction of the left eye? 0 if it is not, 1 if it is.
12. Left Gaze Direction X: The X component of the gaze direction vector for the left eye.
13. Left Gaze Direction Y: The Y component of the gaze direction vector for the left eye.
14. Left Gaze Direction Z: The Z component of the gaze direction vector for the left eye.
15. Right Gaze Direction Validity: Is the eye tracker effectively tracking the gaze direction of the right eye? 0 if it is not, 1 if it is.
16. Right Gaze Direction X: The X component of the gaze direction vector for the right eye.
17. Right Gaze Direction Y: The Y component of the gaze direction vector for the right eye.
18. Right Gaze Direction Z: The Z component of the gaze direction vector for the right eye.
19. Left Gaze Origin Validity: Is the eye tracker effectively tracking the gaze origin of the left eye? 0 if it is not, 1 if it is.
20. Left Gaze Origin X: The X component of the gaze origin point for the left eye.
21. Left Gaze Origin Y: The Y component of the gaze origin point for the left eye.
22. Left Gaze Origin Z: The Z component of the gaze origin point for the left eye.
23. Right Gaze Origin Validity: Is the eye tracker effectively tracking the gaze origin of the right eye? 0 if it is not, 1 if it is.
24. Right Gaze Origin X: The X component of the gaze origin point for the right eye.
25. Right Gaze Origin Y: The Y component of the gaze origin point for the right eye.
26. Right Gaze Origin Z: The Z component of the gaze origin point for the right eye.

ParticipantActions

This stream contains the trigger pulls of the participant, with one channel per controller. These 2 channels are designated right and left. The stream is sampled only when a trigger is pulled, and both controllers are sampled when either one of the triggers are pulled.

Channels:
1. Left Trigger: This channel is true when the left trigger has been pressed, and false otherwise
2. Right Trigger: This channel is true when the right trigger has been pressed, and false otherwise

HeadPose

This stream contains the position and rotation information for the Head Mounted Display in Unity (Virtual Environment) coordinates. The sample rate is the same as the framerate of the Virtual Environment, and so can vary due to changes in simulation load.

Channels:
1. X Position: The X position in meters of the HMD in virtual space
2. Y Position: The Y position in meters of the HMD in virtual space
3. Z Position: The Z position in meters of the HMD in virtual space
4. X Rotation: The rotation in degrees of the HMD around the X axis in virtual space
5. Y Rotation: The rotation in degrees of the HMD around the Y axis in virtual space
6. Z Rotation: The rotation in degrees of the HMD around the Z axis in virtual space

SessionEvent

This stream contains a single channel over which events during the trial(s) are recorded. Currently, the available event markers are "start" when a participant starts a trial, "stop" when the trial ends, and messages denoting that a target has been indicated, presented, or deactivated.

Channels:
1. Event text: A session event, as text

TargetNumericalData

This stream contains all of the numerical information about targets. Samples are taken at the same rate as the Frames per second of the simulation. However, samples are only taken during the target cycle. So, if no targets are active, then no samples will be taken. When a target begins the presentation cycle (turns yellow by default) then this stream will start taking samples, and will continue taking samples until the target completes its cycle and returns to normal. There is more relevant information about the target, but it is formatted as a string through the TargetOtherData stream.

Channels:
1. Position X: The X position in meters of the target in virtual space.
2. Position Y: The Y position in meters of the target in virtual space.
3. Position Z: The Z position in meters of the target in virtual space.
4. Rotation X: The target's rotation around the X axis in degrees.
5. Rotation Y: The target's rotation around the Y axis in degrees.
6. Rotation Z: The target's rotation around the Z axis in degrees.
7. Distance to Participant: How far away the participant is from this target, in virtual space (meters)
8. Target Delay: How long between when the target is indicated and the bullseye is presented. This is set at the beginning of each presentation, and should not change over the course of the target cycle.

TargetOtherData

This stream contains the string formatted information about the target that is currently presenting. As with TargetNumericalData, this stream only takes samples during the target cycle.

Channels:
1. Name: The name of the active target.
2. Size: The size of the active target. This is either 'Small', 'Medium', or 'Large'.
3. Eye Distance: Which eye distance angle was this target placed at? I.e. the distance this target is from the center. Either 'Small', 'Medium', or 'Large'.
4. Target State: What state is the target in? This changes over the course of the target cycle from 'indicated' to 'presenting'.
5. Bullseye Facing: Which side of the bullseye 'O' will open when the target state changes to 'presenting'. Either left or right.

SessionSettings

The Session Settings stream only takes a sample at the start of a trial. This sample is a single string containing the comma separated values that were used in the settings for the trial. This string is in a json format. For more detail about individual settings, see Menu options.

Channels:
1. Settings text: All of the experiment settings in a string, labeled, and comma separated. (json format)

GazeTargetNumericalInfo

The gaze target info takes a sample every time the Tobii Eye Tracker takes a sample. Position, rotation, and distance are recorded for any virtual object the participant looks at. If the participant is staring off into space or the gaze ray is invalid for whatever reason, this stream

will take a sample with 0 for the position and rotation values, and -1 for the distance value. Other relevant information about the object the participant is looking at is accessible from the GazeTargetOtherInfo stream.

Channels:
1. Position X: The X position in meters of the object in virtual space.
2. Position Y: The Y position in meters of the object in virtual space.
3. Position Z: The Z position in meters of the object in virtual space.
4. Rotation X: The object's rotation around the X axis in degrees.
5. Rotation Y: The object's rotation around the Y axis in degrees.
6. Rotation Z: The object's rotation around the Z axis in degrees.
7. Distance to Participant: How far away the participant is from this object, in virtual space (meters)

GazeTargetOtherInfo

The samples of this stream line up with the samples in GazeTargetNumericalInfo to completely define what the participant is looking at. If the gaze ray is invalid, then the samples of this stream will have the value 'None'.

Channels:
1. Name: The name of the object that the participant is currently looking at.

GazeData

This stream contains information about the participant's gaze and how it relates to the virtual world. If the gaze is invalid, then all channels will be zero.

Channels:
1. Gaze Validity: 0 if the current gaze stream is invalid, 1 if it is valid.
2. Gaze Point Position X: The X position of the gaze point in Unity coordinates (meters)
3. Gaze Point Position Y: The Y position of the gaze point in Unity coordinates (meters)
4. Gaze Point Position Z: The Z position of the gaze point in Unity coordinates (meters)
5. Gaze Point Relative Position X: The X position of the gaze point relative to the center of the object the participant is looking at.
6. Gaze Point Relative Position Y: The Y position of the gaze point relative to the center of the object the participant is looking at.
7. Gaze Point Relative Position Z: The Z position of the gaze point relative to the center of the object the participant is looking at.
8. Distance to Participant: How far away the participant is from the gaze point, in unity coordinates (meters)

AngleDeltas

This stream contains the difference in angle of the eyes from the most recent valid sample to the current valid sample. Only valid samples are used, which is why this channel has a variable sample rate. If any of the angle deltas are invalid, no sample will be pushed.

Channels:
1. Left eye angle delta: The difference in degrees between the last valid gaze direction vector for the left eye and the current vector.
2. Right eye angle delta: The difference in degrees between the last valid gaze direction vector for the right eye and the current vector.
3. HMD angle delta: The difference in degrees between the last valid forward vector for the HMD and the current vector.
4. Unity gaze vector angle delta: The difference between the last valid gaze direction for the Unity gaze vector and the current vector.
